# Higher Plasma LDL-Cholesterol is Associated with Preserved Executive and Fine Motor Functions in Parkinson’s Disease

**DOI:** 10.14336/AD.2015.1030

**Published:** 2016-05-27

**Authors:** Nicholas W. Sterling, Maya Lichtenstein, Eun-Young Lee, Mechelle M. Lewis, Alicia Evans, Paul J. Eslinger, Guangwei Du, Xiang Gao, Honglei Chen, Lan Kong, Xuemei Huang

**Affiliations:** ^1^Departments of Neurology, Pennsylvania State University-Milton S. Hershey Medical Center, Hershey PA 17033, USA.; ^3^Pharmacology, Pennsylvania State University-Milton S. Hershey Medical Center, Hershey PA 17033, USA.; ^2^Public Health Sciences, Pennsylvania State University-Milton S. Hershey Medical Center, Hershey PA 17033, USA.; ^4^Radiology, Pennsylvania State University-Milton S. Hershey Medical Center, Hershey PA 17033, USA.; ^5^Neurosurgery, Pennsylvania State University-Milton S. Hershey Medical Center, Hershey PA 17033, USA.; ^6^Kinesiology, Pennsylvania State University-Milton S. Hershey Medical Center, Hershey PA 17033, USA.; ^7^Department of Nutritional Sciences, the Pennsylvania State University, University Park, PA 16802, USA.; ^8^Epidemiology Branch/Aging & Neuroepidemiology Group, National Institute of Environmental Health Sciences, Research Triangle Park, NC 27709, USA

**Keywords:** Parkinson’s disease, cholesterol, low density lipoprotein cholesterol (LDL-cholesterol), cognition, neuroprotection, executive function

## Abstract

Plasma low density lipoprotein (LDL) cholesterol has been associated both with risk of Parkinson’s disease (PD) and with age-related changes in cognitive function. This prospective study examined the relationship between baseline plasma LDL-cholesterol and cognitive changes in PD and matched Controls. Fasting plasma LDL-cholesterol levels were obtained at baseline from 64 non-demented PD subjects (62.7 ± 7.9 y) and 64 Controls (61.3 ± 6.8 y). Subjects underwent comprehensive neuropsychological testing at baseline, 18-, and 36-months. Linear mixed-effects modeling was used to assess the relationships between baseline LDL-cholesterol levels and longitudinal cognitive changes. At baseline, PD patients had lower scores of fine motor (p<0.0001), executive set shifting (p=0.018), and mental processing speed (p=0.049) compared to Controls. Longitudinally, Controls demonstrated improved fine motor and memory test scores (p=0.044, and p=0.003), whereas PD patients demonstrated significantly accelerated loss in fine motor skill (p=0.002) compared to Controls. Within the PD group, however, higher LDL-cholesterol levels were associated with improved executive set shifting (β=0.003, p<0.001) and fine motor scores (β=0.002, p=0.030) over time. These associations were absent in Controls (p>0.7). The cholesterol - executive set shifting association differed significantly between PDs and Controls (interaction p=0.005), whereas the cholesterol - fine motor association difference did not reach significance (interaction, p=0.104). In summary, higher plasma LDL-cholesterol levels were associated with better executive function and fine motor performance over time in PD, both of which may reflect an effect on nigrostriatal mediation. Confirmation of these results and elucidation of involved mechanisms are warranted, and might lead to feasible therapeutic strategies.

Parkinson’s disease (PD) is a common age-related neurodegenerative disorder marked pathologically by Lewy pathology and the death of dopamine neurons in the substantia nigra pars compacta. Although the cardinal manifestations of PD are related to motor symptoms, cognitive decline often is present, even in early-stage disease, and this can be severely disabling in later stages [1]. Twenty years after diagnosis, dementia affects more than 80% of PD patients [2].

Previous research has suggested an age-dependent relationship between plasma cholesterol and cognition throughout the lifespan. For example, higher plasma cholesterol in mid-life (age ~50 years) has been associated with higher risk of dementia and mild cognitive impairment many years later [3, 4], whereas higher cholesterol in late-life (age ~70-80 years) has been associated with better cognitive function and lower risk of dementia [5-7]. Similarly, several studies have suggested that higher total- and/or LDL-cholesterol levels may be associated with lower risk and beneficial outcomes in PD. For example, three recent case-control studies suggested that higher plasma cholesterol levels may be associated with lower occurrence of PD [8-10]. In addition, four independent prospective studies indicated that higher plasma cholesterol may be associated with lower future risk of PD [11-14], although one prospective study reported an opposite association [15]. Higher LDL-cholesterol also has been associated with a trend of slower motor symptom progression in PD [16]. Most recently, a longitudinal study demonstrated that idiopathic rapid eye movement sleep behavior disorder patients with hypercholesterolemia are less likely to convert to dementia with Lewy bodies or PD [17]. These findings raise the possibility that there may be a beneficial relationship between higher plasma LDL-cholesterol and PD.

There are no known previous studies investigating cognition and cholesterol in PD, but we hypothesized that higher baseline LDL-cholesterol would be associated with slower cognitive decline in PD participants. This was tested by examining the association between baseline plasma cholesterol levels and prospective changes in cognitive scores over 36 months in PD subjects and matched Controls.

## MATERIALS AND METHODS

### Participants

Based on criteria outlined below, 64 PD subjects and 64 Controls who had completed blood collection and cognitive testing were selected from an ongoing longitudinal cohort study of 70 PD and 70 Control subjects using nearest neighbor propensity scoring for age, gender, and dropout rate over 36 months [18]. PD diagnosis was confirmed by an experienced movement disorders specialist according to published criteria [19]. Controls were recruited from spouses and from the local community. All subjects were deemed to be free of acute medical issues and major neurological disease (other than PD) by reviewing full medical histories, medication lists, and laboratory data that included chemistry panels, liver enzyme tests (aspartate transaminase and alanine transaminase), creatinine, and thyroid stimulating hormone. Subjects having a Mini Mental Status Examination (MMSE) score <26 at baseline were excluded from the analysis [20]. Written informed consent was obtained for all subjects and the study was conducted in accordance with the Declaration of Helsinki. The research study protocol and procedures were reviewed and approved by the Penn State Hershey Institutional Review Board.

**Table 1:** Demographics of study subjects at baseline (averages presented as mean ± SD)

	PD, n=64	Control, n=64	P-values
n, Female : n, Male	26 : 38	32 : 32	0.374
Statin Use (No : Yes)	46 : 18	49 : 15	0.686
Smoker (No : Yes)	48 : 16	49 : 15	1.000
Age (years)	62.7 ± 7.9	61.3 ± 6.8	0.268
Education (years)	15.6 ± 2.8	16.9 ± 2.6	***0.008***
LDL-cholesterol (mg/dL)	127.3 ± 33.0	126.5 ± 41.3	0.616
MMSE	29.3 ± 1.0	29.5 ± 0.9	0.698
Hamilton Depression Scale	7.7 ± 4.8	4.0 ± 2.6	***<0.0001***
LEDD (mg)	709 ± 481	NA	-
Disease duration (years)	4.4 ± 4.4	NA	-
Hoehn & Yahr Score	1.7 ± 0.7	NA	

Abbreviations - MMSE: Mini Mental Status Examination; LEDD: Levodopa equivalent daily dosage; LDL: Low density lipoprotein

### Blood cholesterol, exposures, and potential confounding factors

Blood was collected at baseline after an 8-12 hour overnight fast. Total plasma cholesterol and triglycerides were measured by enzymatic methods as described previously using an Ortho Vitros 4600 [21]. LDL-cholesterol was calculated using the Friedwald equation: LDL_C_ = Chol_total_ - (Triglycerides/5 + HDL_C_). Statin use (yes/no) and factors that might influence cognition (age, gender) were recorded at baseline. Education level was recorded as total years of schooling. Cigarette smoking information was obtained as part of a comprehensive demographic survey. Subjects were considered to be smokers (yes/no) if they had smoked one cigarette per day for at least six months [22]. Depression was considered as a continuous variable at each visit and symptoms of depression assessed using the Hamilton depression scale (HAM-D) [23].

### Neuropsychological examinations

A comprehensive neuropsychological battery was administered at baseline, 18-, and 36-month visits (Table 2). For PD subjects, this was done in a practically defined “off” state following overnight withdrawal from PD medications, to minimize the influence of anti-Parkinson’s disease drugs [24].

As detailed below, the battery of neuropsychological tests assessed eight cognitive domains: (i) fine motor speed; (ii) memory; (iii) executive function (spontaneous flexibility); (iv) executive function (set shifting); (v) attention/working memory; (vi) processing speed; (vii) language; and (viii) spatial cognition. Cognitive domain scores were defined as the mean of the population z-scores of individual cognitive tests (Table 2).

*Fine motor* scores were calculated as the mean time of the dominant and non-dominant hands using the Grooved Pegboard test [25]. *Memory* was assessed using the Brief Visuospatial Memory Test [26] and Hopkins Verbal Learning Test [27]. *Executive functions requiring spontaneous flexibility* were evaluated using the design and verbal fluency tests of the Delis-Kaplan Executive Function System. *Executive functions requiring set shifting* were assessed using the color-word interference test of the Delis-Kaplan Executive Function System [28]. *Attention/working memo*ry was measured by letter number sequencing, spatial span, and digit span tests from the Wechsler Memory Scale-III [29]. The assessment of *processing speed* was based on scores from the color naming portion of the color-word interference test of the Delis-Kaplan Executive Function System [28], and the symbol search test of the Wechsler Adult Intelligence Scale IV [30]. *Language* was evaluated using the word reading portion of the color-word subtest of the Delis-Kaplan Executive Function System [28] and the Boston Naming Test [31]. *Spatial cognition* was assessed using the Judgment of Line Orientation test [32].

**Table 2:** Individual cognitive tests comprising cognitive domains

Cognitive Domain	Individual Tests
Fine motor speed	Grooved Pegboard Test
Memory	Brief Visuospatial Memory Test-Revised (BVMT-R)
	Hopkins Verbal Learning Test-Revised (HVLT-R)
Executive function: Spontaneous flexibility	DKEFS Design Fluency Test (DesFlu)
	Verbal Fluency Test (VerbFlu)
Executive function: Set-shifting	CWInt-Switch and CWInt-Inhibition subtests, including error scores
Attention//Working memory	Digit Span
	Spatial Span
	Letter-Number Sequencing Test
Processing speed	CWInt color
	Symbol search
Language	Boston Naming Test (BNT)
	CWInt-Word subtest
Spatial cognition	Benton’s Judgment of Line Orientation (JoLO)

### Statistical analysis

Demographic information, clinical characteristics, and neuropsychological domain z-scores were compared between PD and Control subjects using one-way analysis of covariance (ANCOVA) with adjustment for age, gender, and education as appropriate. Gender was compared between groups using Fisher’s exact tests. Cross-sectional comparisons of individual cognitive scores between PD and Control subjects were performed using ANCOVA with age, gender, and education level as covariates. At baseline, multiple linear regression analysis was used to evaluate the relationships between cholesterol level and cognition, adjusting for age, gender, education, statin usage, levodopa daily equivalent dosage (LEDD), and PD status, using the following interaction terms: 1) statin usage × cholesterol, and 2) PD status × cholesterol levels.

Longitudinal analyses of cognitive change over time were performed using linear mixed-effects modeling. The group difference was examined before and after the adjustment of other covariates of interest, respectively. The mixed-effects model to investigate the correlations between baseline cholesterol levels and cognitive change included random slopes and intercepts, with cognitive score as the dependent variable and the following independent variables: age at baseline, years elapsed (since baseline), gender, LEDD, group, education years, depression score, statin usage, and cholesterol level. The interaction terms included the following: 1) statin usage × cholesterol level, 2) group × years elapsed, 3) years elapsed × cholesterol level, 4) group × cholesterol level, and 5) years elapsed × group × cholesterol level. The coefficient associated with the three-way interaction (years elapsed × group × cholesterol level) indicates whether the association of lipid and rate of cognitive change was different between PD and Controls. The association between cholesterol and cognition was estimated for each group (PD and Controls) and tested using F-testing of the fixed effects. Due to the stepwise nature of this analysis, all statistical results are reported as raw p-values. Results that survived adjustment for multiple comparisons using the Bonferroni method across eight tests are noted [33]. All analyses were completed using R version 3.1.1.

## RESULTS

### Characteristics of subjects

As shown in Table 1, there were no differences in age or gender frequencies between PD and Controls at baseline. PD subjects, however, had fewer years of education (p=0.008). There were no significant group differences in cholesterol levels, statin usage, or MMSE scores. The average disease duration for the PD group at baseline was 4.4 ± 4.4 y (mean ± SD). The average Hoehn & Yahr score was 1.7 ± 0.7.

**Table 3:** Association of baseline LDL-cholesterol levels with change in cognitive function over time (using baseline, 18-month, and 36-month scores)

	PD	Control	P-values
**Baseline Visit**			
Fine Motor Speed	-2.03 ± 1.11	-0.46 ± 1.08	***<0.001***
Memory	-0.59 ± 0.91	-0.45 ± 0.92	0.403
Executive Function: SF	0.11 ± 0.78	0.43 ± 0.70	0.090
Executive Function: SS	-0.01 ± 0.94	0.44 ± 0.47	***0.018***
Attention	0.29 ± 0.661	0.37 ± 0.53	0.803
Processing Speed	0.08 ± 0.57	0.29 ± 0.32	***0.049***
Language	0.29 ± 0.80	0.51 ± 0.49	0.246
Spatial Cognition	-0.27 ± 0.93	-0.19 ± 0.77	0.787
**18-Months Visit**			
Fine Motor Speed	-2.03 ± 1.06	-0.31 ± 0.98	***<0.001***
Memory	-0.60 ± 0.81	-0.24 ± 0.80	0.067
Executive Function: SF	0.00 ± 0.87	0.27 ± 0.73	0.095
Executive Function: SS	0.01 ± 0.88	0.46 ± 0.41	***0.008***
Attention	0.19 ± 0.75	0.36 ± 0.55	0.456
Processing Speed	-0.02 ± 0.59	0.23 ± 0.31	***0.022***
Language	0.19 ± 0.76	0.54 ± 0.48	***0.015***
Spatial Cognition	-0.18 ± 0.91	-0.15 ± 1.00	0.610
**36-Months Visit**			
Fine Motor Speed	-2.09 ± 1.09	-0.19 ± 0.97	***<0.001***
Memory	-0.45 ± 0.92	-0.05 ± 0.74	0.098
Executive Function: SF	-0.08 ± 0.73	0.37 ± 0.76	***0.022***
Executive Function: SS	0.21 ± 0.74	0.52 ± 0.47	0.059
Attention	0.33 ± 0.59	0.34 ± 0.56	0.949
Processing Speed	0.08 ± 0.56	0.21 ± 0.38	0.300
Language	0.32 ± 0.86	0.64 ± 0.44	0.053
Spatial Cognition	-0.11 ± 0.59	-0.13 ± 0.83	0.952

* Significant after correction for multiple comparisons. Abbreviations: SF: Spontaneous flexibility; SS: Set shifting.

Overall, PD subjects showed higher depression scores at each visit (p≤0.001) compared to Controls. We also compared characteristics of subjects who remained in the study and those who were lost to follow-up. For both cases and Controls, there were no statistical differences in baseline LDL-cholesterol, total-cholesterol, age, gender, education years, MMSE and cognitive scores between subjects who remained in the study and those lost to follow-up.

### Neuropsychological scores in PD and Controls

Cross-sectional analyses showed that PD subjects performed significantly worse than Controls in fine motor speed skill at all three visits (p<0.0001). PD subjects also demonstrated lower performance in executive set shifting and processing speed at baseline and 18 months (p<0.05), and tended to have lower executive set shifting function at 36 months (p=0.059). Full details of cognitive scores at each visit are summarized in Table 3.

Longitudinal analysis indicated that Controls showed improved fine motor (p=0.044) and memory (p=0.003) performance over time, perhaps due to practice effects and adaptation to the testing [34-36]. No other cognitive domain demonstrated changes within the Control group. PD subjects, however, had worsening performance in fine motor (p=0.009), processing speed (p=0.013), executive spontaneous flexibility (p=0.015) and attention/working memory (trend-level, p=0.054) tasks over time.

Compared to Controls, PD subjects showed accelerated loss of fine motor speed skill (p=0.002) and a trend of accelerated loss of memory (p=0.066) and language (p=0.080). PD and Control subjects, however, had no significant differences in rate of executive set shifting changes (p=0.937).

### Correlation between cholesterol and cognition at baseline

In Controls, there were no significant associations between baseline cholesterol and cognitive scores. Within PD subjects, higher LDL cholesterol was associated with lower language scores at baseline (β=0.008, p=0.015).

### Relationship between baseline LDL-cholesterol and longitudinal cognitive changes

**Figure 1.**
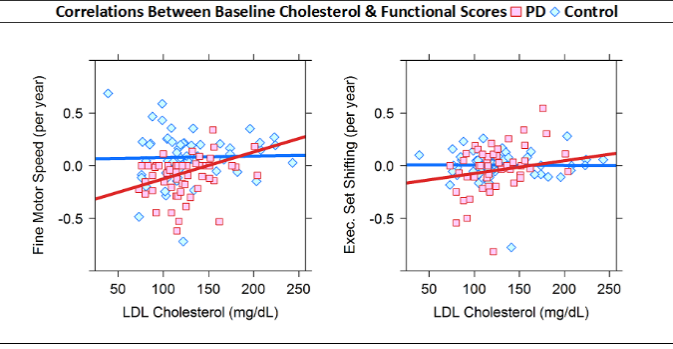
Annual rates of changes in cognitive function over time associated with baseline plasma LDL-cholesterol levels Annual rates of change estimated using linear regression of baseline, 18-month, and 36-month cognitive z-scores.

Longitudinal analysis demonstrated that higher baseline LDL-cholesterol was significantly associated with improved executive set shifting scores over time in the PD group (β=0.003, p<0.001), whereas this association was not seen in Controls (see Figure 1). There was a significant three-way interaction between baseline LDL-cholesterol level, group, and years elapsed related to executive set shifting (β=0.003, p=0.005). This indicated that the predictive association of LDL-cholesterol level and rate of change in executive set shifting was significantly different between PD and Controls (Table 4, Figure 1).

**Table 4:** Association of baseline LDL-cholesterol levels with change in cognitive function over time (using baseline, 18-month, and 36-month scores)

	PD		Controls		Group Difference	
	**Estimate**	**P-Value**	**Estimate**	**P-Value**	**Estimate**	**P-Value**
Fine motor speed	0.002	0.030	0.000	0.801	0.002	0.104
Memory	0.001	0.289	0.001	0.394	0.001	0.726
Executive function: SF	0.000	0.741	0.000	0.482	0.000	0.900
Executive function: SS	0.003	<0.001*	0.000	0.905	0.003	0.005*
Attention	0.001	0.500	0.001	**0.035**	-0.001	0.526
Processing speed	0.000	0.742	0.000	0.811	0.000	0.889
Language	0.001	0.240	0.000	0.967	0.001	0.348
Spatial cognition	0.001	0.563	0.000	0.875	0.001	0.705

* Significant after correction for multiple comparisons. Abbreviations: SF: Spontaneous flexibility; SS: Set shifting.

Higher baseline LDL-cholesterol was significantly associated with improved fine motor speed skill over time in the PD group (β=0.002, p=0.030), whereas this association was absent in Controls. The three-way interaction analysis between baseline LDL-cholesterol level, group, and years elapsed (since baseline) related to fine motor speed skill did not reach statistical significance (β=0.002, p=0.104). This indicated that the predictive association of LDL-cholesterol level and rate of change in fine motor speed skill did not differ significantly between PD and Controls.

### Influence of statin use on cholesterol-cognition relationship

To assess potential modifying effects of statin use in the longitudinal cholesterol-cognitive relationship in PD, we performed a sensitivity analysis by stratifying subjects according to baseline statin use. Average (mean ± SD) baseline LDL-cholesterol in PD *statin non-users* was 136 ± 33.1 mg/dL and 105 ± 20.2 mg/dL in PD *statin users*. Average (mean ± SD) baseline LDL-cholesterol in Control *statin non-users* was 134 ± 38.7 mg/dL and 100 ± 40.0 mg/dL in Control *statin users*.

Among PD *statin non-users*, higher baseline LDL-cholesterol continued to be associated significantly with improved executive set shifting (β=0.004, p<0.001) and fine motor speed (β=0.003, p=0.030) scores over time. This association was not observed in Control *statin non- users*. In addition, the significant group differences in cholesterol-cognition associations persisted for executive set shifting (interaction β=0.003, p=0.002), but not in fine motor speed (interaction β=0.002, p=0.283). This indicated that the association of LDL level on the rate of change in executive set shifting (but not fine motor speed) was significantly different between PD and Control statin non-users.

Among *statin users*, there was no statistically significant difference between PD and Controls regarding LDL-cholesterol-cognition associations for executive set shifting (interaction β=0.004, p=0.195) or fine motor scores (interaction β=0.004, p=0.269). In PD statin users, baseline LDL-cholesterol had no association with fine motor (β=0.003, p=0.277) or executive set shifting over time (β=0.003, p=0.206). Interestingly, there was a positive association between higher baseline LDL- cholesterol and improvement in language performance over time in PD-statin users (β=0.003, p=0.036). No associations between baseline LDL-cholesterol and cognition over time were observed in Control statin users.

## DISCUSSION

The current study provided the first evidence that in non-demented PD subjects, higher plasma LDL-cholesterol may be associated prospectively with better executive set shifting and fine motor function longitudinally. These cholesterol-cognitive relationships were specific to PD and not found in the matched Controls. These findings are consistent with previous studies suggesting beneficial outcomes in PD [16] and lower risk [11-14] with plasma higher LDL-cholesterol. Future studies are warranted to investigate the mechanisms underlying these relationships.

In the current study, baseline LDL-cholesterol was associated prospectively with better performance in cognitive domains that are more specifically affected in PD (e.g., executive set shifting and fine visuomotor speed). Each mg/dL increase in LDL-cholesterol was associated with a z-score increase of 0.003 per year in the executive set shifting task. According to these results, a baseline LDL-cholesterol difference of 20 mg/dL could be extrapolated to predict an increase or decrease in the executive set shifting z-score of 0.3 over five years (20 mg/dL x 0.003/year x 5 years). Recent studies, for example, have suggested that performance reduction in z-scores by 1 or 1.5 in more than 2 subtests or domains could be meaningful clinically to define MCI or predict future conversion to dementia [37]. Thus, the cholesterol-cognitive association in PD may be meaningful clinically over the span of several years in terms of development of dementia.

Although the LDL-cholesterol relationship may be etiological (*vide supra*), it is possible that this relationship could be driven by behavioral factors associated with the disease (i.e. nutritional and/or lifestyle). Even if the cholesterol-cognition association is causal, careful risk-benefit analyses would be needed to establish the appropriate cholesterol target and therapeutic choices given the well-known adverse effects of LDL-cholesterol on cardiovascular disease.

It is interesting to note that cognitive domains associated with cholesterol in PD have been correlated with striatal functioning. For example, lower performance on the Grooved Pegboard test is associated closely with nigrostriatal denervation [38]. Similarly, frontostriatal dysfunction in PD results in poor performance on set-shifting tasks [39]. This raises the possibility that the interaction between cholesterol-cognition might occur at the level of nigrostriatal function. Although speculative, there are several supportive data. For example, higher plasma LDL-cholesterol levels are associated with markers of lower iron accumulation in the substantia nigra [40]. It also has been reported that higher cholesterol in late-life (age ~70-80 years) is associated with better cognition in healthy populations [5-7], and this may be relevant because impairment of nigrostriatal integrity is known to occur in the normal aging process, although at a much slower rate compared to PD subjects [41]. Together, these results raise the possibility that the association between cholesterol and cognition may be nigrostriatal-based.

Although the exact biological basis of the cholesterol-cognition relationship is unclear, we speculate that one possibility is that cholesterol could facilitate compensatory repair of injured neuronal pathways in PD. Indeed, cholesterol is necessary for synaptogenesis, and antagonizing the LDL receptor disrupts this process [42, 43]. In normal brain, however, cholesterol is synthesized primarily by astrocytes and then is transported to neurons via endocytosis and interaction with the LDL receptor and apolipoprotein E [42]. There is limited ability for plasma cholesterol to transverse the blood brain barrier [44]. Thus, brain cholesterol levels are made mainly *de novo*, and might not be linked directly to plasma cholesterol level [45]. In PD, some reports have suggested an increased cholesterol content in cortices [46], and increased levels of cholesterol breakdown products in the cerebrospinal fluid of patients [47, 48]. Together, these data may suggest that brain cholesterol turnover is increased in PD. It will be very interesting to determine if the ability of plasma cholesterol to traverse the blood-brain barrier is changed in the disease condition. On the other hand, if the cholesterol in the CNS and periphery are highly compartmentalized, it may be that the effects of cholesterol are indirect (e.g., reflecting toxicant metabolism or transport).

In our sensitivity analysis, we examined the effect of baseline statin use on the cholesterol-cognitive correlations in PD. Consistent with the results of the main analysis, baseline LDL-cholesterol was correlated with better executive set shifting and fine motor performance over time in *statin non-users*. It is interesting to note that these correlations were not extended to *statin users*. This later finding may be related to a lower amount of variance in baseline plasma levels and a smaller sample size in the PD *statin users* subgroup than in the PD *statin non-users* subgroup. There have been suggestions that statins are neuroprotective in PD, although this is controversial [49, 50]. Future studies with bigger sample sizes and detailed and stratified analyses of statin effects on PD progression are warranted.

### Strengths and limitations

The strengths of the study included: 1) prospective data analysis with both 18- and 36-month follow-up; 2) comprehensive cognitive batteries to evaluate domain-specific cholesterol-cognitive associations; and 3) inclusion of controls of similar age ranges that enabled us to find the differential cholesterol-cognitive relations between PD and Controls. Major limitations include: 1) the study design could not tease out if the cholesterol-cognitive relationship is causative, reverse causative, or a parallel process; 2) the sample size was relatively small as this is the first explorative study of the relationship between cholesterol and cognition. Nevertheless, the association of cholesterol with executive set shifting in PD was strong enough to survive correction for multiple comparisons; and 3) the study could not tease out the effects of statin subgroups on PD progression.

### Summary

To our knowledge, this is the first study investigating the relationship between LDL-cholesterol and cognitive function in PD subjects. The results indicated that higher LDL-cholesterol in PD is associated with better performance in functional domains that depend on proper nigrostriatal functioning (i.e. fine motor speed skill and executive function). Although the LDL-cholesterol relationship may be etiological (*vide supra*), it also is possible that this relationship could be driven by behavioral factors associated with the disease (i.e., nutritional, medical, and/or lifestyle). Similarly, reduced plasma cholesterol may serve simply as a warning sign and may not be connected directly to brain cholesterol metabolism. Even if the cholesterol-cognition association is causal, careful risk-benefit analyses would be needed to establish the appropriate cholesterol target and therapeutic choices given the well-known adverse effects of LDL-cholesterol on cardiovascular disease. Future studies are warranted to confirm these findings and explore potential mechanisms.

This work was supported by the National Institute Neurological Disorders and Stroke (NS060722 and NS082151 to XH), the Hershey Medical Center GCRC (National Institute of Health M01RR10732), the GCRC Construction Grant (C06RR016499), the Pennsylvania Department of Health Tobacco CURE Funds, and the intramural research program of the National Institute of Environmental Health Sciences (Z01-ES-101986). All analyses, interpretations, and conclusions are those of the authors and not the research sponsors.
